# From scales to circuits: integrating behavioral diagnosis and neural biomarkers for improved classification in disorders of consciousness

**DOI:** 10.3389/fnins.2025.1725420

**Published:** 2025-12-18

**Authors:** Shanshan Chen, Lubin Wang, Yituo Wang, Zheng Yang

**Affiliations:** 1 Xinjiang Key Laboratory of Mental Development and Learning Science, Urumqi, Xinjiang, China; 2 School of Psychology, Xinjiang Normal University, Urumqi, Xinjiang, China; 3 The Brain Science Center, Beijing Institute of Basic Medical Sciences, Beijing, China; 4 Department of Radiology, The Seventh Medical Center of Chinese PLA General Hospital, Beijing, China

**Keywords:** disorders of consciousness, machine learning, minimally conscious state, resting-state fMRI, unresponsive wakefulness syndrome

## Abstract

**Introduction:**

In this study, we propose a data-driven approach that integrates behavioral diagnosis with neuroimaging features to identify representative UWS and MCS patients from a large inpatient cohort.

**Methods:**

Clinical information was extracted using a subset of UWS patients with CRS-R scores ≤ 5. Neuroimaging biomarkers were established as the increased and decreased functional connectivity indices of anatomically defined regions covering the whole brain. The algorithm was implemented through an iterative refinement process that converged on a division of UWS and MCS patients into representative and excluded (or nonrepresentative) patient groups.

**Results:**

Thirty-one out of 58 UWS patients and 23 out of 30 MCS patients were identified as representative, with an average classification accuracy of 90.2% in differentiating between the two groups. In contrast, differentiating between excluded UWS patients (*n* = 27) and representative MCS patients (*n* = 23) and between all UWS (*n* = 58) and MCS (*n* = 30) patients produced average classification accuracies of 50.9 and 64.3%, respectively. Furthermore, altered DMN functional connectivity between representative UWS and MCS patients revealed a consistent pattern as shown in prior studies, while comparisons involving excluded patients did not.

**Discussion:**

These results highlight the value of integrating behavioral scores and neural connectivity features for DOC classification, providing a more coherent basis for downstream analysis and machine-learning applications in DOC classification.

## Introduction

Differentiating patients with unresponsive wakefulness syndrome (UWS), also known as the vegetative state (Laureys et al., 2010) from those in a minimally conscious state (MCS) remains one of the most challenging clinical tasks in the diagnosis, prognosis, and treatment of patients with disorders of consciousness (DOC) (Giacino et al., 2014; Xiao et al., 2024). Clinical evaluation of the level of consciousness in unresponsive and minimally conscious patients is demanding because of the presence of motor impairment, sensory deficit, altered cognition, fluctuation of vigilance or arousal level, and medical complications in patients as well as examiner bias (Schnakers, 2012; Wannez et al., 2017). The misdiagnosis rate is worryingly high and reaches up to 40% if it is based exclusively on clinical consensus (Gill-Thwaites, 2006; Schnakers et al., 2009; Stender et al., 2014; Wang et al., 2020; Porcaro et al., 2022).

Neuroimaging techniques provide an opportunity to gain a deeper understanding of altered brain functions underlying states of diminished consciousness and can complement the clinical assessment to improve the diagnosis and prognosis of patients with DOC (Gosseries et al., 2016; Alnagger et al., 2023; Wang et al., 2024). Recently, neuroimaging technologies, such as EEG and functional MRI, in combination with artificial intelligence (AI) offer promising solutions to enhance diagnostic accuracy in DOC patients (Calderone et al., 2024; Velasco-Muñoz et al., 2025). AI-based methods, particularly machine learning models including support vector machine (SVM), convolutional neural network model and graph neural network model have been demonstrated holding promise in improving classification accuracy of DOC states (Noirhomme et al., 2017; Campbell et al., 2020; Kim et al., 2024; Qi et al., 2024; Yang et al., 2024).

Despite considerable progress in combining neuroimaging and machine learning to assist in diagnosis and prognosis of DOC, an inherent limitation in the establishment of the computational classifier models lies in the fact that the supervised machine learning techniques rely on the prerequisite of correctly labeled/diagnosed patient samples (Noirhomme et al., 2017). When training deep learning models, correct data labeling play a critical role in determining model performance (Hao et al., 2020). However, in the DOC domain, mislabeling is particularly problematic due to the high misdiagnosis rate (~40%) (Schnakers et al., 2009; Giacino et al., 2014). Such errors may arise from clinician subjectivity, patient arousal fluctuations, or insufficient diagnostic repetitions (Schnakers et al., 2009; Van Erp et al., 2015; Wannez et al., 2017; Golden et al., 2024), thereby introducing label noise into the training set. The presence of mislabeled patients impairs not only the training of machine learning-based classifiers but also the subsequent interpretation of classifier results (Zhang et al., 2016; Noirhomme et al., 2017). Given the high-rate mislabeled characteristic of DOC dataset, identifying representative or prototypical patient subgroups prior to model training is essential. This strategy could facilitate the construction of reliable training subsets, mitigate the impact of mislabeled or atypical cases, and enhance both the stability and generalizability of machine learning models applied in this domain.

The goal of this study was to propose a novel approach for identifying representative UWS and MCS patients (UWS: 64; MCS: 30; Table 1) who were behaviorally diagnosed with the Coma Recovery Scale-Revised (CRS-R) (Giacino et al., 2004) and were assigned clinical labels (i.e., UWS or MCS) on the day the resting-state fMRI was performed. In our proposed approach, the clinical diagnostic information was initially extracted or represented using the subset of UWS patients with CRS-R scores ≤ 5. We consider that the misdiagnosis rate is considerably low for UWS patients with low CRS-R scores (i.e., ≤5). Neuroimaging biomarkers were established as the increased and decreased functional connectivity indices (defining a two-dimensional feature space) derived from a network consisting of 116 standard anatomical regions (Tzourio-Mazoyer et al., 2002) as regions of interest (ROIs) or network nodes covering the whole brain. Briefly, our approach was implemented by an iterative refinement process similar to the expectation–maximization algorithm, as commonly seen in iterative algorithms such as the k-means clustering. The core algorithm involves comparing healthy control subjects (*n* = 20) and selected UWS patients updated at each iterative step, while projecting the rest of the excluded (not selected) UWS and MCS patients in the feature space. In a few iterations, the algorithm converges, dividing UWS and MCS patients into representative and excluded (or nonrepresentative) patient groups. We argue that a combination of clinical diagnostic information and neuroimaging biomarkers provides a better way to identify correctly labeled or representative UWS and MCS patients than the clinical behavioral assessment alone.

**Table 1:** Demographic and clinical characteristics of patients.

Patient ID	Gender	Age	Months since onset	Etiology	Lesion information	CRS-R score (1st)	Diagnosis
1	M	23	5	TBI	Right basal ganglia lesion	7	MCS
2	M	23	6	TBI	DAI lesion and left thalamus and brainstem atrophy	9	MCS
3	F	28	1	TBI	Brainstem atrophy	15	MCS
4	F	31	3	TBI	Left thalamus and brainstem atrophy	11	MCS
5	M	60	7	TBI	Diffuse white matter damage	11	MCS
6	M	58	12	TBI	Bilateral frontal contusion and brainstem hemorrhage	8	MCS
7	M	29	9	TBI	Multi-focal contusion	18	MCS
8	M	41	1	TBI	DAI and subcortical atrophy	11	MCS
9	F	27	10	TBI	Multi-focal contusion and left frontoparietal hemorrhage	12	MCS
10	F	27	10	TBI	DAI lesion	13	MCS
11	M	39	1	HI	Left basal ganglia hemorrhage and brain atrophy	17	MCS
12	M	23	3	HI	Brainstem atrophy	10	MCS
13	F	28	6	Poisoning	Anoxia caused by drug poisoning	9	MCS
14	F	28	9	Poisoning	Anoxia caused by drug poisoning	10	MCS
15	F	29	21	Poisoning	Anoxia caused by drug poisoning	11	MCS
16	M	61	2	HI	Left basal ganglia and left thalamus hemorrhage	11	MCS
17	M	42	3	HI	Brain atrophy	7	MCS
18	M	42	2	HI	Left thalamus hemorrhage	8	MCS
19	M	53	7	HI	Right basal ganglia hemorrhage	11	MCS
20	M	45	2	HIE	Anoxia caused by drug poisoning	9	MCS
21	M	45	9	HI	Brainstem hemorrhage	9	MCS
22	M	32	6	HI	Multi-focal hemorrhagic lesion	11	MCS
23	M	28	2	CPA	n/a	9	MCS
24	M	28	5	CA	n/a	8	MCS
25	M	46	2	CPA	n/a	9	MCS
26	M	51	6	CA	n/a	9	MCS
27	M	45	3	HIE	n/a	10	MCS
28	F	25	3	HIE	n/a	10	MCS
29	M	41	3	HIE	n/a	11	MCS
30	M	36	5	TBI	n/a	10	MCS
31	M	61	9	TBI	Right frontal contusion	6	VS
32	F	46	15	TBI	n/a	7	VS
33	F	58	2	TBI	Left parietal-occipital lesion and brainstem hemorrhage	3	VS
34	F	66	1	TBI	Bilateral frontoparietal lesion	6	VS
35	M	23	10	TBI	Right frontal lesion	7	VS
36	M	46	4	TBI	Right temporal, parietal and occipital lesion	6	VS
37	M	39	5	TBI	Right basal ganglia hemorrhage	7	VS
38	M	34	3	TBI	DAI and brain atrophy	6	VS
39	M	22	3	TBI	Left frontal, temporal and parietal lesions	7	VS
40	M	51	3	TBI	Right frontal, temporal and parietal lesions	7	VS
41	M	45	1	TBI	n/a	6	VS
42	F	68	6	TBI	Brainstem and right basal ganglia lesions	7	VS
43	F	64	1	HI	Left thalamus and basal ganglia hemorrhage	7	VS
44	M	46	2	HIE	Brainstem hemorrhage	6	VS
45	M	52	4	HIE	Brainstem hemorrhage	5	VS
46	M	39	3	HIE	Left basal ganglia hemorrhage	7	VS
47	F	53	3	HIE	Brainstem hemorrhage	5	VS
48	M	49	10	HI	Brainstem lesion	7	VS
49	M	45	3	TBI	Diffuse white matter damage	7	VS
50	F	67	4	HI	Bilateral brain atrophy	6	VS
51	F	40	5	HI	Brainstem hemorrhage	7	VS
52	M	58	6	HI	Right temporal lobe hemorrhage	7	VS
53	F	26	4	HI	Left basal ganglia hemorrhage	6	VS
54	F	69	4	HI	Multi-focal cerebral infarction	6	VS
55	F	60	4	HIE	Anoxia caused by anesthesia	6	VS
56	M	42	6	HI	Right hemisphere and brainstem lesions	7	VS
57	M	36	1	HIE	Multi-focal cerebral infarction	7	VS
58	M	48	4	HIE	Anoxia caused by electric shock	7	VS
59	F	28	5	HIE	n/a	6	VS
60	M	40	4	HI	Brainstem hemorrhage	4	VS
61	F	35	3	HIE	Severe brain atrophy	6	VS
62	F	29	28	Eclampsia	Intraparenchymal hemorrhage and severe brain atrophy	7	VS
63	F	28	3	HIE	Brainstem lesion and brain atrophy	6	VS
64	F	35	3	HIE	Bilateral basal ganglia and thalamus lesions	7	VS
65	F	42	1	CA	Anoxia caused by amniotic fluid embolism	7	VS
66	M	43	2	CA	Cardiac arrest caused by coronary heart diseases	5	VS
67	F	51	6	CPA	n/a	6	VS
68	M	51	3	CPA	n/a	7	VS
69	F	35	2	CPA	Bilateral frontoparietal lesions	7	VS
70	F	38	2	CPA	Basal ganglia and frontal, temporal and parietal lesions	6	VS
71	M	41	2	CPA	Multi-focal cerebral infarction	5	VS
72	F	50	8	CPA	Bilateral basal ganglia lesions and brain atrophy	6	VS
73	F	71	30	CPA	Multi-focal cerebral infarction and brain atrophy	7	VS
74	M	52	2	CA	n/a	7	VS
75	F	50	6	CPA	n/a	5	VS
76	M	45	9	HIE	n/a	5	VS
77	M	33	9	HIE	n/a	5	VS
78	F	73	2	HIE	n/a	7	VS
79	M	62	2	HIE	n/a	3	VS
80	M	30	2	HIE	n/a	6	VS
81	M	18	8	Asphyxia	n/a	6	VS
82	F	71	3	HIE	n/a	9	VS
83	F	57	2	HIE	n/a	5	VS
84	M	26	54	HIE	n/a	6	VS
85	F		2	CPA	n/a	n/a	VS
86	F	79	2	HIE	n/a	6	VS
87	M	33	2	HIE	n/a	5	VS
88	M	71	6	TBI	Right tempo-parietal lesion and DAI	3	VS

## Materials and methods

This study was approved by the ethics committee of the Seventh Medical Center of Chinese PLA General Hospital. Written informed consent was obtained from healthy volunteers and from the legal surrogate of each patient.

### Study participants

Study participants included 64 patients in UWS, 34 patients in MCS, and 25 age-matched normal control subjects enrolled in Beijing Army General Hospital from 2013 to 2016. At the time of enrollment, patients had remained in UWS or MCS for at least 1 month after severe brain injuries. Table 1 summarizes the clinical profiles of the enrolled patients. Patients were excluded if they had any of the following clinical conditions: (1) a history of drug or alcohol abuse, (2) a history of psychiatric or neurological illness, or (3) were under sedation or anesthesia during fMRI acquisition. The level of consciousness in patients was assessed using the CRS-R (Giacino et al., 2004). For each patient, the first one-time CRS-R assessment was made and diagnostic labeling (i.e., UWS or MCS) was assigned on the day resting-state fMRI took place. Six UWS patients and four MCS patients were excluded due to excessive head motion during functional imaging or excessive deformation in brain structure. Five healthy control subjects were excluded due to head motion and falling asleep during scans. Application of the exclusion criteria resulted in 58 UWS patients, 30 MCS patients, and 20 healthy control subjects included in the final data analysis.

### Data acquisition

Structural and resting-state fMRI data were acquired using a whole-body 3 T Signa GE scanner (GE Healthcare, Waukesha, WI, USA) with a standard transmit-receive head coil. Functional imaging blood-oxygen-level-dependent (BOLD) signals were obtained during a 7-min scan for each subject using the following sequence parameters: repetition time, 2 s; echo time, 25 ms; total volumes, 210; slice thickness, 4.0 mm; in-plane resolution, 3.75 × 3.75 mm; number of slices, 39; flip angle, 90°; field of view, 24.0 cm; matrix size, 64 × 64. High-resolution three-dimensional spoiled gradient-recalled echo axial images were acquired before functional scans with TE/TR/TI, 3.2/8.2/450 ms; slice thickness, 1 mm; 188 slices; flip angle, 7°; field of view, 256 mm; matrix size, 256 × 256.

### Data preprocessing

Imaging data preprocessing was conducted using a combination of AFNI (Analysis of Functional NeuroImages, 2017 release),^1^ SPM12 (Statistical Parametric Mapping)^2^ and Matlab (MathWorks, R2017b). For each subject, head-motion quality was examined using SPM (Realign). Subjects whose translational displacement exceeded 3 mm in any direction were excluded from the final dataset. T1-weighted anatomical images of each subject were first manually transformed into the standard Talairach space; then each subject’s functional imaging data were registered into the Talairach space with a resampling to a 3-mm cubic voxel size. The first four functional data points were discarded to reduce the initial transient effects in data acquisition. Subsequent data preprocessing included slice timing correction (3dTshift in AFNI), despiking (3dDespike in AFNI), and motion correction (3dvolreg in AFNI, producing three translational and three rotational parameters for each volume image). Physiological noise was estimated using the average BOLD fMRI signals from regions of white matter and CSF determined in each individual’s anatomical images in the Talairach space. The voxelwise BOLD fMRI signals were then analyzed with a general linear regression model (3dDeconvolve in AFNI) using the eight regressors representing noise artifacts from the motion parameters, white matter, and cerebrospinal fluid, respectively. The residual voxelwise time courses from the regression analysis were considered as the resting-state BOLD fMRI signals with potential noise contaminations minimized. The denoised functional images were then transformed into the MNI (Montreal Neuroimaging Institute) space (MNI152; 3dWarp in AFNI) with a 3-mm cubic voxel size. In the MNI space, functional data were further cleaned by regressing out artifacts originating from subregions of major vein (e.g., superior sagittal sinus) areas. The cleaned voxelwise BOLD fMRI signals were bandpass filtered to preserve only the amplitude of low-frequency fluctuations within 0.01–0.1 Hz for the subsequent functional connectivity analysis.

### Patient classification using 116-ROI-based functional connectivity matrices

Neuroimaging biomarkers for differentiating between any two target groups (i.e., control vs. UWS/MCS or UWS vs. MCS) were established as the *decreased connectivity index* (DCI) and the *increased connectivity index* (ICI) derived from a 116-ROI-based functional connectivity matrix for each subject. The segmentation of the brain into 116 anatomical regions was obtained using a standard brain atlas (Tzourio-Mazoyer et al., 2002) in the MNI space. For each subject, the mean time series of preprocessed BOLD fMRI signals of all voxels included in each of the 116 anatomical regions was computed as the representative ROI or network node time series. Functional connectivity was then assessed by Pearson’s correlation coefficients of representative ROI time series among all the 116 ROIs, resulting in a symmetric 116-by-116 functional connectivity matrix with 6,670 (116 × 115/2) distinct node pairs. The correlation coefficients were normalized using the Fisher transformation, and the functional connectivity matrices were compared between any two target groups by two-sample *t*-tests (*p* < 0.05).

In contrasting functional connectivity matrices between any two groups (e.g., control vs. UWS), we defined the DCI as the mean of the correlation coefficients of node pairs whose functional connectivity strength significantly decreased in the second group (e.g., UWS) relative to the first group (e.g., control). Likewise, the ICI was defined as the mean of the correlation coefficients of nodes pairs whose functional connectivity strength significantly increased in the second group (e.g., UWS) relative to the first group (e.g., control). The DCI and ICI together define a two-dimensional feature space, in which machine learning techniques can be applied to differentiate between target groups.

In all cases of this study, we used a support vector machine with a linear kernel function to classify between any two groups of healthy control subjects and patients. Because of the availability of large samples in identified groups, we divided the overall data set of each group into training and test sets to overcome the potential problem of overfitting. The assignment of members to the training and test sets was randomly repeated 500 times. We report the distribution of classification accuracies from the 500 repetitions as well as the mean values of classification accuracy for each analysis.

### An expectation–maximization-algorithm-like iterative refinement process for identifying the representative UWS and MCS patients

To identify representative UWS and MCS patients, we developed an iterative expectation–maximization-like (EM-like) refinement procedure operating in the two-dimensional DCI-ICI feature space.

1 Objective function: The refinement was formalized as minimizing a Mahalamobis-distance-based function that minimizes within-group Mahalanobis distance while maximizing between-group separation:

$$L=\sum_{i\in \mathrm{UWS}}{d_{M\;(\mathrm{xi},\text{μUWS}\;)}}^{2}+\sum_{i\in \mathrm{HC}}{d_{M\;(\mathrm{xi},\mathrm{\mu HC}\;)}}^{2}-\lambda {d_{M\;(\text{μUWS},\mathrm{\mu HC}\;)}}^{2}$$

Where

- 𝑥_𝑖_ denotes the feature vector of patient 𝑖;
- *μ*_𝑘_ is the is the centroid of group 𝑘
- 𝑑_𝑀_ is the Mahalanobis distance using group covariance *Σ*𝑘
- *λ* controls the trade-off between intra- and inter-cluster terms.

2 Confidence Ellipses: For each group, a confidence ellipse in the feature space was computed using:

$${\left(x-\mu \right)}^{T}\Sigma^{-1}(x-\mu )\leq \chi_{0.9,\mathrm{df}=2}^{2}\;$$

Where $\chi_{0.9,2}^{2}$ = 4.605, so that the semi-axes are scaled by $\surd \chi_{0.9,2}^{2}$ ≈ 2.147 times the standard deviations along the principal directions. Thus, the ‘90% ellipse’ is not arbitrary but corresponds to the standard 90% confidence region of a bivariate Gaussian distribution in the DCI–ICI feature space.

3 E-step: Updating membership

In each iteration, the membership of UWS patients was updated by:

- computing the Mahalanobis distance of each patient to the current UWS centroid;
- determining whether the patient’s feature vector falls inside the statistically defined confidence ellipse;
- updating the membership list of “included” and “excluded” UWS patients accordingly.

This step corresponds to an EM-style expectation (E) step, where latent membership labels are iteratively reassigned.

4 M-step: Updating group statistics

Given updated membership assignments, the following parameters were recomputed:

- group means 𝜇𝑈𝑊𝑆 and 𝜇𝐻𝐶,
- covariance matrices *Σ*𝑈𝑊𝑆 and Σ𝐻𝐶,
- the corresponding confidence ellipses.

This corresponds to the EM-style maximization (M) step, updating the model parameters conditioned on new label assignments.

**Figure 1:**
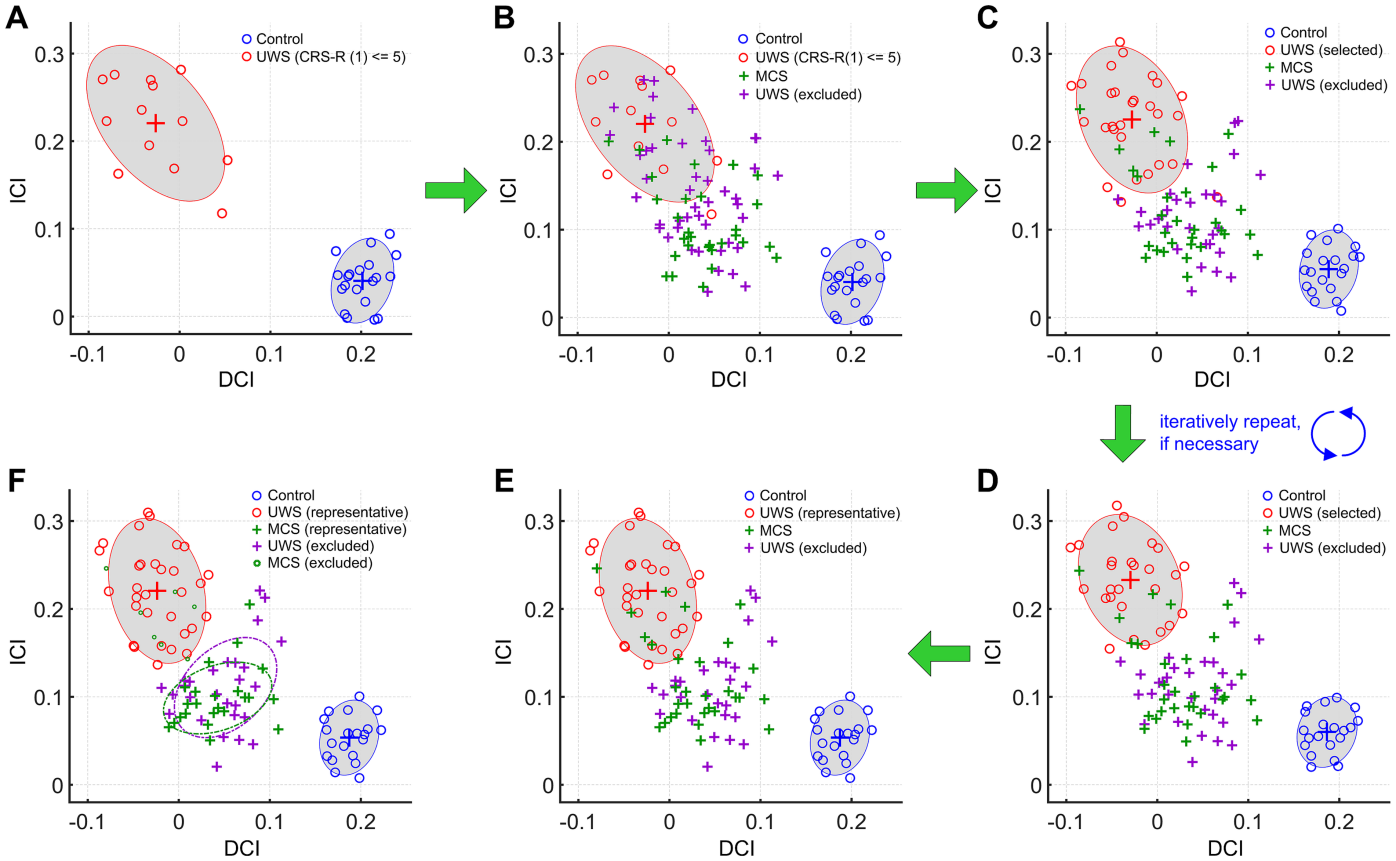
The iterative refinement process for the identification of representative UWS and MCS patients. In all steps, feature vectors defined by [DCI, ICI] were calculated by contrasting the 116-ROI-based functional connectivity matrices of the selected UWS patients with those of 20 healthy control subjects. **(A)** The distribution of feature vectors of the first selected UWS patients with CRS-R scores ≤ 5 and 20 healthy control subjects in step 1. **(B)** Overlaying feature vectors of excluded UWS patients and all MCS patients in the plot shown in panel **(A)**. **(C)** The distribution of feature vectors of selected and excluded UWS patients, all MCS patients, and healthy control subjects produced by the second iteration of the algorithm. The selected UWS patients were assembled by selecting all UWS patients whose feature vectors fall within the 90% probability containment ellipses and removing those whose feature vectors fall outside the ellipses as shown in panel **(B)**. **(D)** The distribution of feature vectors of all groups after repeating the iterative procedures twice from the results shown in panel **(C)**. No feature vectors of excluded UWS patients fall within the red containment ellipses of the selected UWS patients. **(E)** The distribution of feature vectors of all groups after fine-tuning the membership of the excluded UWS patients whose feature vectors are located near the red ellipses in panel **(D)**. Spatial separation between the feature clusters of the selected and excluded UWS patients was optimized. **(F)** The conclusive results of identified patient groups based on the criteria specified in step 6.

5 Convergence criteria: Convergence was defined using three mathematically explicit criteria, in addition to the operational rule illustrated in Figure 1:

- Label stability criterion: No change in membership between iterations.
- Centroid stability criterion: $∥\mu {\left(t\right)}^{\left(t\right)}-\mu {\left(t-1\right)}^{\left(t-1\right)}∥<10^{-4}$.
- Maximum-iteration safeguard: Based on empirical evaluation, the algorithm consistently stabilized within 2–3 iterations; therefore, a maximum of three iterations was allowed.

6 Operational implementation of the refinement algorithm: The algorithm can be summarized in six steps:

- Start by identifying the subgroup of UWS patients with CRS-R scores ≤ 5. Contrast the 116-ROI-based functional connectivity matrices of the selected UWS patients with those of 20 healthy control subjects. Compute the DCI and ICI accordingly for each subject. Plot the distribution of feature vectors (defined by [DCI, ICI]) in the feature space for all subjects in both groups (Figure 1A; red circles represent selected UWS patients and blue circles represent control subjects) and draw the estimated 90% probability containment ellipses of feature vectors for each group (Figure 1A).
- Plot feature vectors ([DCI, ICI]) of all excluded UWS patients (i.e., those with CRS-R scores > 5, purple crosses in Figure 1B) and all MCS patients (green crosses in Figure 1B) in the feature space. Note that the DCI and ICI of the excluded patients were computed using the significant node pairs identified in step 1. Next, determine the identities of excluded UWS patients whose feature vectors fall within the 90% probability containment ellipses of the selected UWS patients with CRS-R scores ≤ 5 (Figure 1B).
- Reassemble a set of UWS patients by selecting all UWS patients whose feature vectors fall within the 90% probability containment ellipses and removing those whose feature vectors fall outside the ellipses. Compare the functional connectivity matrices of the reassembled UWS patients with those of healthy control subjects. Compute the DCI and ICI for all subjects in the same way as in steps 1 and 2. Plot feature vectors and 90% probability containment ellipses for UWS and control subjects, as well as feature vectors of excluded UWS and MCS patients (Figure 1C).
- Repeat step 3 until no feature vectors of excluded UWS patients fall within the 90% probability containment ellipses of the selected UWS patients (Figure 1D).
- Fine-tune the membership of the excluded UWS patients whose feature vectors are located near the red ellipses until a desired separation between included UWS patients (circumscribed by the red ellipses) and excluded UWS patients is formed (Figure 1E).
- Consider UWS patients circumscribed by the red ellipses as representative (red circles in Figure 1F) and those falling outside as excluded (or nonrepresentative; purple crosses in Figure 1F). Likewise, consider MCS patients whose feature vectors fall outside of the red ellipses as representative (green crosses) and those inside of the red ellipses as excluded (or nonrepresentative; green circles in Figure 1F).

It should be noted that the clinical diagnosis information was incorporated by the procedures taken in step 1 (i.e., by first selecting the subset of UWS patients with CRS-R scores ≤ 5). We consider that misdiagnosis is less likely to occur in UWS patients whose CRS-R scores are considerably low. In this way, the clinical diagnosis information was integrated in the algorithm. In addition, fine-tuning the membership of UWS patients in step 5 does not significantly alter the final results; it only leads to slight changes to the lists of selected and excluded patients. For step 3, we found that it usually took two to three iterations for our results to converge.

### Validation

We validated the identified patient groups in two ways. First, we compared the classification performance of a support-vector-machine classifier in differentiating between (1) the identified representative UWS and MCS patients, (2) the excluded UWS patients and representative MCS patients, and (3) all UWS and MCS patients with the original clinical labels. Classification performance for separating representative UWS and MCS patients should rank the highest. Second, we examined the DMN functional connectivity in healthy control subjects, identified UWS and MCS groups using the PCC as the seed region, and compared the results to prior studies. Individual group or between-group DMN connectivity maps were obtained by one- and two-sample *t*-tests (*p* < 0.05) with correction of multiple comparisons using a probability and cluster thresholding technique (*AlphaSim* in AFNI; a minimum cluster size > 3,960 mm^3^). To demonstrate the performance of the proposed network analysis, defined features, and the classifier in differentiating patients from healthy control subjects, we also examined the classifier’s performance in differentiating all undivided UWS (*n* = 58) and MCS (*n* = 30) patients from healthy control subjects (*n* = 20), respectively. We included this part of the results in Supplementary information.

## Results

### Representative and excluded UWS and MCS patients

Among the 58 UWS patients included in data analysis, 13 have CRS-R scores ≤ 5 (Figure 1A; red circles). These 13 UWS patients were used to represent clinical diagnosis information in step 1 of our algorithm. Feature vectors defined by [DCI, ICI] of the 13 UWS patients and 20 healthy control subjects were acquired by contrasting the 116-ROI-based functional connectivity matrix between the two groups. The distribution of feature vectors of the two groups formed two well-spatially separated clusters in the feature space (Figure 1A). It is noticeable that the distribution of feature vectors of the UWS patients was relatively more widespread than that of the healthy control subjects, indicating a higher degree of inter-subject homogeneity of functional connectivity measurements in healthy control subjects than in UWS patients. The feature vector from one UWS patient was located outside the 90% probability containment ellipses and in the direction of the feature cluster of healthy control subjects (Figure 1A), indicating that the UWS patient should be removed from the selected list in the next iteration of the algorithm.

Overlaying feature vectors of the excluded UWS patients and all MCS patients on top of Figure 1A revealed a sizable portion of excluded UWS patients (*n* = 18) whose feature vectors were located within the 90% probability containment ellipses of the selected UWS patients (Figure 1B; purple crosses within the red ellipses). The observation indicates that these 18 UWS patients exhibit similar functional connectivity features (i.e., the definition of DCI and ICI) to those of the 13 UWS patients with CRS-R scores ≤ 5. Accordingly, those UWS patients were included in the selected list (for comparison with 20 healthy control subjects) in the next iteration step of the algorithm. In addition, feature vectors from eight MCS patients were also located within the containment ellipses of the selected UWS patients (Figure 1B; green crosses within the red containment ellipses), suggesting those MCS patients are similar to the selected UWS patients in terms of the defined functional connectivity features. The feature vectors of the rest of the MCS patients were widely distributed between the feature clusters of the selected UWS patients and healthy control subjects, spatially mixed with those of many excluded UWS patients (Figure 1B).

The UWS patients were reassembled for comparison with the control subjects in the next iteration; this was done by selecting from all the UWS patients whose feature vectors were located inside the red ellipses and rejecting the previously selected UWS patients whose feature vectors fell outside of the containment ellipses and toward the feature cluster of control subjects. The second iteration’s comparison produced spatially separated clusters of the two groups with fewer numbers of the excluded UWS patients mixed with the selected ones (Figure 1C). One selected UWS patient still had a feature vector located outside of the red ellipses and toward that of the healthy control subjects. The iterative process performing the described criteria for UWS patient selection and deselection as described in step 3 was repeated twice, producing separated clusters of the selected UWS patients and healthy control subjects with no excluded UWS patients mixed with the selected ones and no UWS patients to deselect (Figure 1D). Fine-tuning the membership (i.e., as selected or excluded) of the excluded UWS patients whose feature vectors were located near the red containment ellipses, we reached the final solution of our iterative algorithm for identifying representative/excluded patient groups (Figures 1E,F). Specifically, 31 out of 58 UWS patients (Figure 1F; red circles) and 23 out of 30 MCS patients (Figure 1F; green crosses) were identified as the representative UWS and MCS patients, respectively. In the feature space, seven MCS patients (Figure 1F; small green circles) were mixed with the representative UWS patients and 27 UWS patients (Figure 1F; purple crosses) were mixed with the representative MCS patients; these patients were considered as excluded MCS and UWS patient groups. After the fine-tuning process, spatial separations among the containment ellipses of the identified groups were optimized, except for the mixed groups.

### Classification performance in differentiating UWS and MCS patients of identified groups

**Figure 2:**
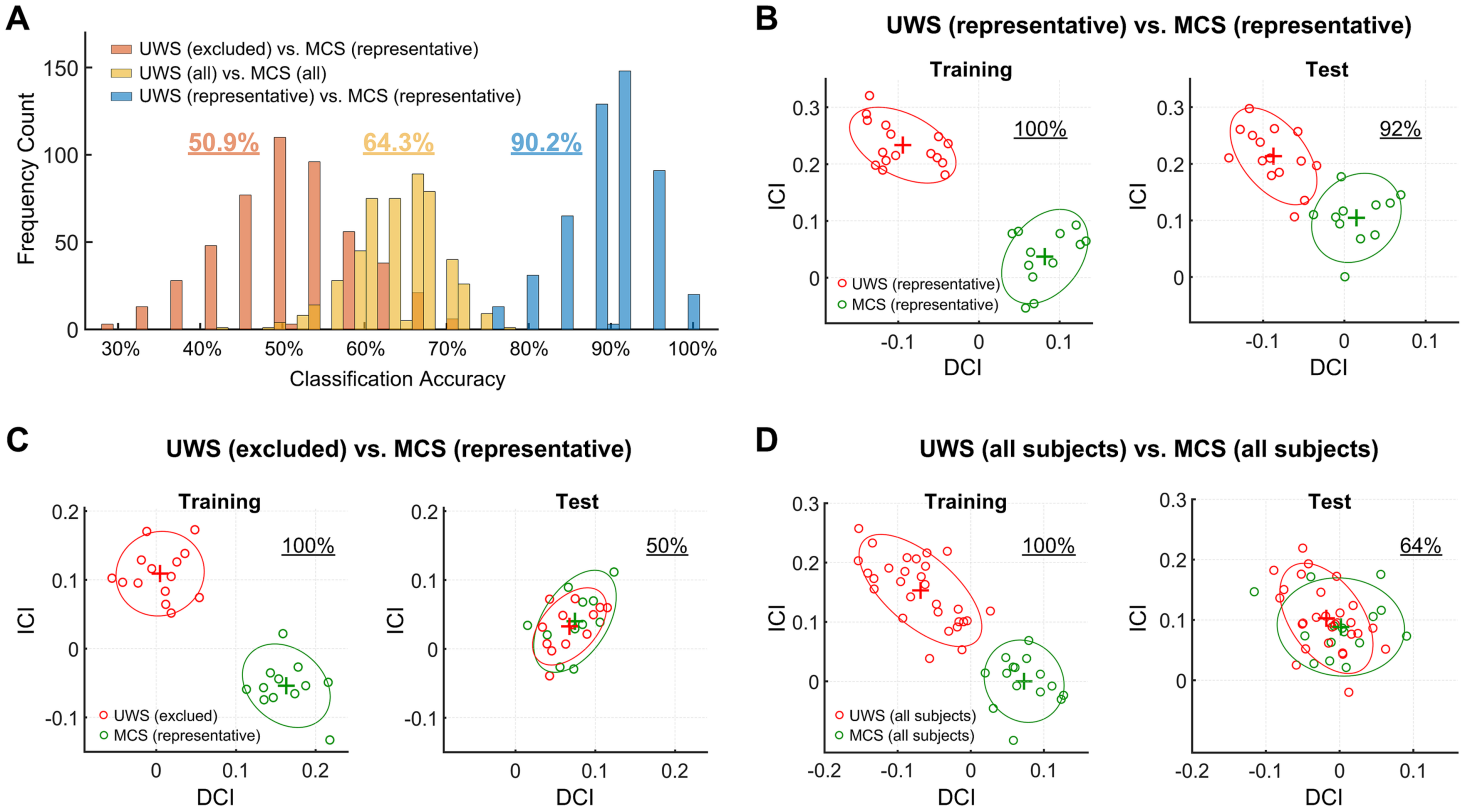
**(A)** The distribution of classification accuracy over 500 repetitions of random division of training and test sets for differentiating between (1) the identified representative UWS and MCS patients, (2) the excluded UWS patients and representative MCS patients, and (3) all UWS and MCS patients. The feature vectors ([DCI, ICI]) were calculated by contrasting the 116-ROI-based functional connectivity matrices of the two groups in each case. The average classification accuracy reached 90.2, 50.9, and 64.3%, respectively, in the three cases. **(B–D)** A representative example of the distribution of training and test samples in the feature space was demonstrated for classification accuracy near the average classification accuracy in each case.

By dividing the data set in each identified group into equally numbered, or near-equally numbered (in case the number of patients is odd), training and test sets, a support-vector-machine classifier was used in the two-dimensional feature space to differentiate between (1) the identified representative UWS and MCS patients, (2) the excluded UWS patients and representative MCS patients, and (3) all UWS and MCS patients with their original clinical labels. For each group, the division of patients into training and test sets was randomly repeated 500 times in each case. Figure 2A illustrates the distribution of classification accuracy over 500 repetitions in the three cases. The average classification accuracy reached 90.2% when differentiating between the representative UWS and MCS patients, 64.3% between all UWS and MCS patients, and 50.9% between the excluded UWS patients and representative MCS patients, respectively (Figure 2A). One representative example of the distribution of training and test samples in the feature space was demonstrated for classification accuracy near the average classification accuracy in each case (Figures 2B–D). A clear between-group separation of test samples was evident for the identified representative UWS and MCS patients (Figure 2B), but this was not the case for the other two conditions (Figures 2C,D). These results indicate that for the identified representative UWS and MCS patients, the classifier built on training samples performed superiorly (~90.2%) on an independent test set, while the classifier for differentiating among the mixed groups of the excluded UWS patients and representative MCS patients in the feature space cannot perform better than chance (~50.9%), and the classifier for differentiating all clinically labeled UWS and MCS patients performed poorly (~64.3%), far below reaching a potential level of clinical significance.

### DMN functional connectivity in healthy control subjects and identified patient groups

**Figure 3:**
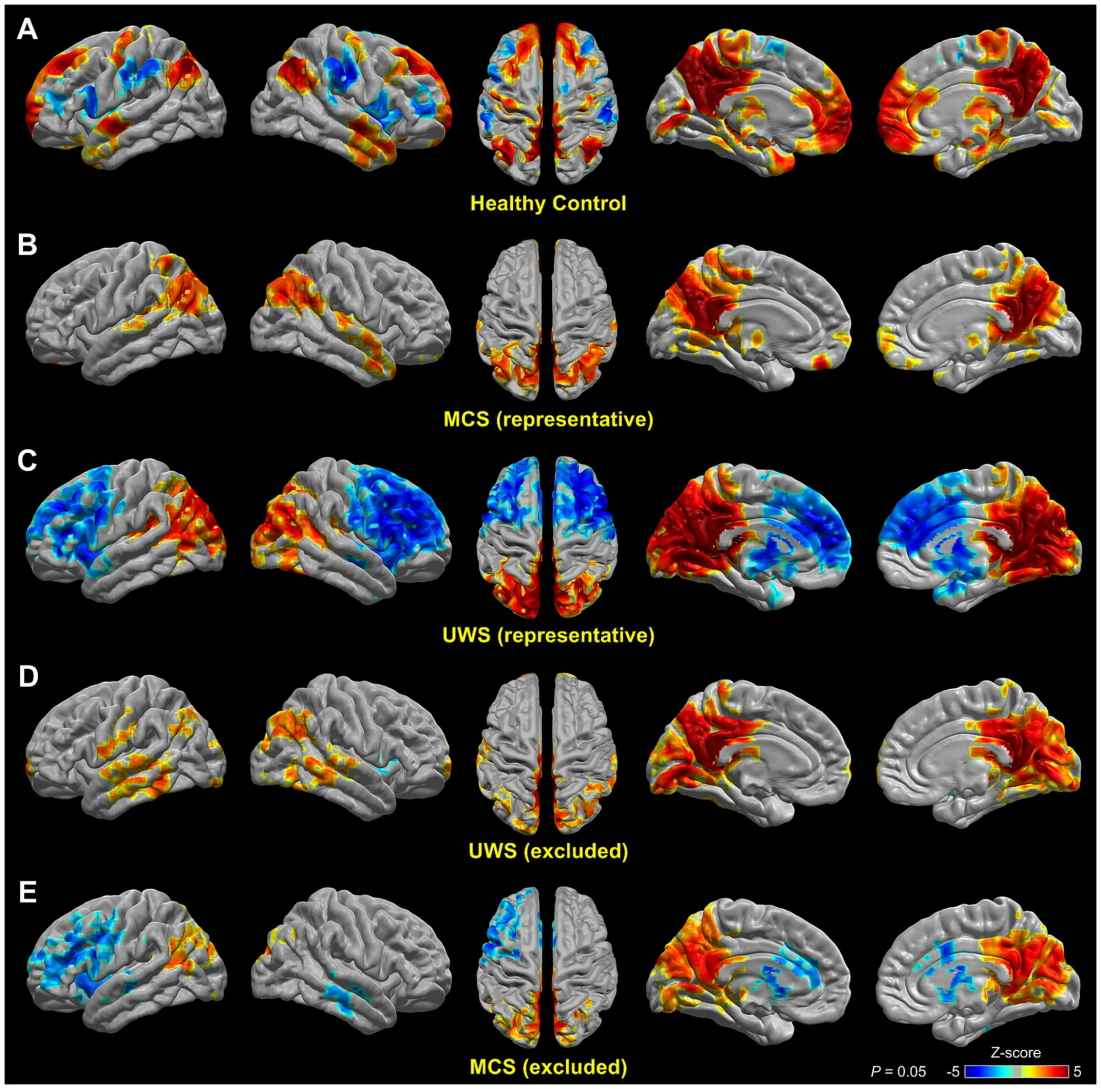
The DMN functional connectivity using the PCC as a seed in healthy control subjects and identified patient groups. Compared with healthy controls subjects **(A)**, the degree to which functional connections between the PCC and the medial and bilateral PFC, medial temporal lobe, and thalamus were reduced deepened from the representative MCS **(B)** to UWS patients **(C)**. The excluded UWS **(D)** and MCS **(E)** patients, however, showed functional connectivity patterns similar to their respective mixed representative MCS and UWS patient groups in the feature space (Figure 1H). The significance of results is reported at *p* = 0.05, corrected for multiple comparisons.

Healthy control subjects exhibited a typical pattern of DMN functional connectivity (Raichle, 2011), with significant positive connections in the PCC and precuneus, ventromedial PFC (vmPFC), bilateral angular gyrus, medial and lateral temporal lobe, and dorsal thalamus, and negative connections in several key regions of the task-positive network, including the dorsal bilateral PFC, supplementary motor area (SMA), and bilateral supramarginal gyrus (Figure 3A). In comparison, the identified representative MCS patients (*n* = 23) exhibited largely reduced PCC–vmPFC connections, with only a few scattered spots of positive connection in the vmPFC (Figure 3B). Moreover, connectivity in the areas of the medial temporal lobe was reduced, and the negative connections with task-positive network regions as observed in healthy control subjects disappeared in representative MCS patients (Figure 3B). In contrast, the identified representative UWS patients (31) exhibited further reduced PCC connections in the medial and bilateral PFC, the medial temporal lobe, and the thalamus relative to the representative MCS patients (Figure 3C); specifically, the PCC indeed showed widespread negative connections with these regions in the representative UWS patients, together with a prominent increase in the PCC connection in the medial and lateral occipital lobe (Figure 3C). As anticipated, the excluded UWS patients (*n* = 27), who were mixed with the representative MCS patients (*n* = 23) in the feature space, showed a DMN connectivity pattern (Figure 3D) similar to that of the representative MCS patients (*n* = 23) in Figure 3B. Likewise, the excluded MCS patients (*n* = 7) showed a DMN connectivity pattern (Figure 3E) similar to that of the representative UWS patients (*n* = 31) in Figure 3C. In summary, the reduction in PCC connectivity with the medial and bilateral PFC, medial temporal lobe, and thalamus became more pronounced from representative MCS to representative UWS patients. The excluded UWS and MCS patients showed DMN functional connectivity patterns similar to their respective mixed representative MCS and UWS patient groups in the feature space.

**Figure 4:**
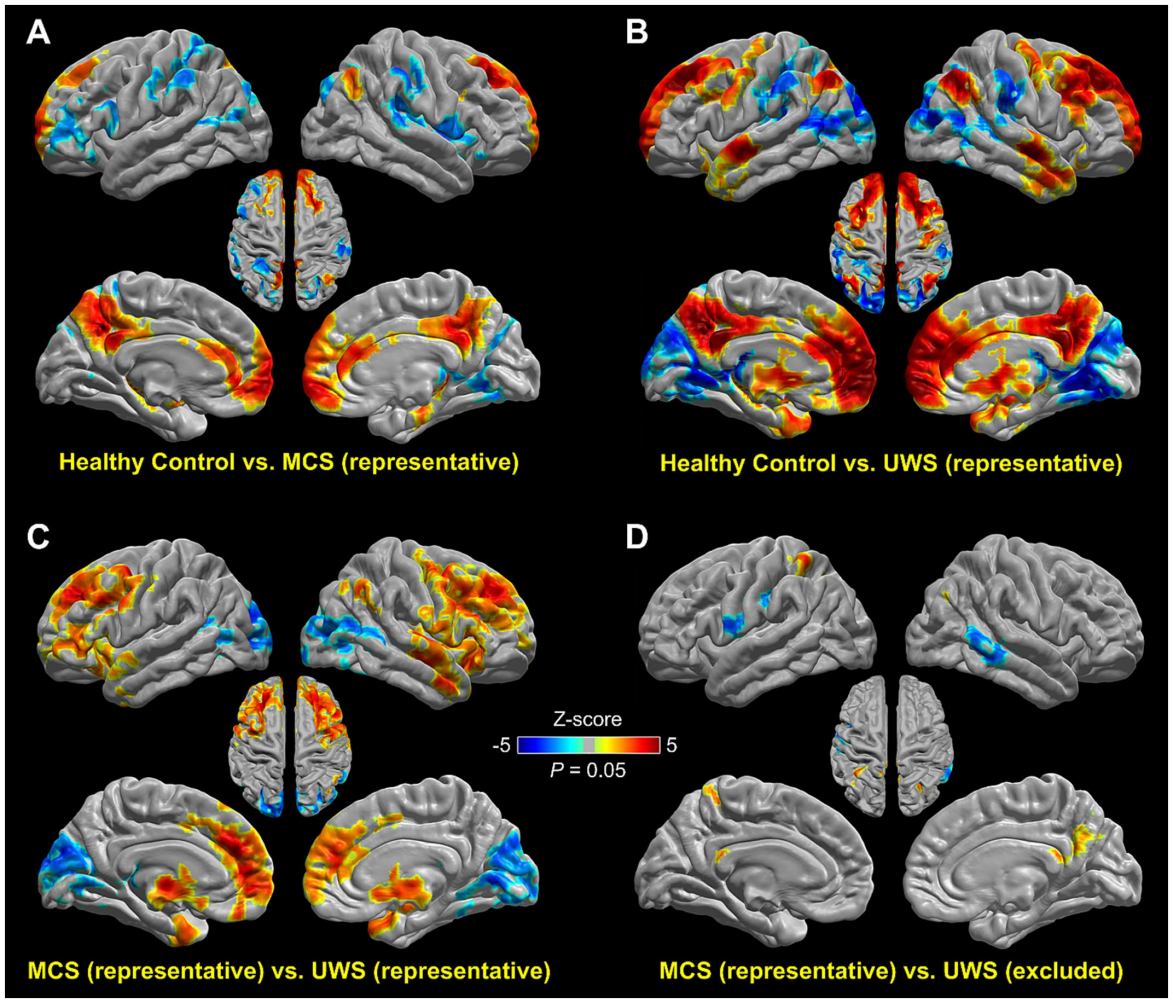
Two-sample *t*-test comparisons of PCC functional connectivity between healthy control subjects and identified patient groups. The representative MCS and UWS patients exhibited a significant reduction of PCC connections in major DMN hubs when compared with healthy control subjects **(A,B)** and each other **(C)**. Patient groups showed increased PCC functional connectivity in the occipital lobe, and the increase enlarged from MCS to UWS. Contrasting representative MCS and excluded UWS patients, however, showed few coherent differences **(D)**. Warm colors indicate higher functional connectivity strength in the first group of each paired comparison. The significance of results is reported at *p* = 0.05, corrected for multiple comparisons.

Group comparisons revealed between-group differences similar to those observed in individual group maps. Compared with control subjects, representative MCS patients showed reduced PCC connections in several major cortical DMN hubs (i.e., the PCC, precuneus, vmPFC, right angular gyrus) but increased connectivity in a few frontal–parietal task-positive network regions (Figure 4A). In contrast, compared with healthy control subjects, representative UWS patients showed reduced PCC connections in all cortical DMN hubs in both hemispheres (Figure 4B), but with the extent of areas and the magnitude of reduction increased prominently when compared with those identified by the comparison between the representative MCS patients and healthy control subjects in Figure 4A. Compared with healthy control subjects, increased PCC connectivity in representative UWS patients was found in the bilateral supramarginal gyrus and occipital lobes but not in the frontal lobes. Consistently, contrasting representative UWS and MCS patients revealed reduced DMN functional connectivity in the medial and bilateral PFC, medial temporal lobe, and thalamus (Figure 4C). However, contrasting representative MCS and excluded UWS patients showed differences only in several scattered spots in the parietal and temporal lobes (Figure 4D). Moreover, contrasting representative UWS (*n* = 31) and excluded MCS (*n* = 7) patients revealed no difference in any areas (Supplementary Figure 2A). Together, these results show that the identified representative MCS and UWS patients exhibited a significant reduction of PCC connections in major DMN hubs when compared with healthy control subjects and each other, while comparisons between representative MCS and excluded UWS patients and between representative UWS and excluded MCS patients showed few coherent or no differences.

### Classification performance in differentiating UWS and MCS from healthy control subjects

Additional analyses examined the classifier’s performance in differentiating all undivided UWS (*n* = 58) and MCS (*n* = 30) patients from healthy control subjects (*n* = 20), respectively. The distributions of classification accuracies (across 500 repetitions) are shown in Supplementary Figures 1A,B, with the average classification accuracy reaching 99.5 and 97.2% in the two cases (Supplementary Figures 1A,B). One representative example of the distribution of training and test samples in the feature space is demonstrated for each case (Supplementary Figures 1C,D). These results suggest a superior performance of the proposed network analysis, defined features, and the classifier in characterizing altered network functional connectivity in UWS and MCS patients relative to healthy control subjects. Because the primary interest of this study focuses on how the proposed approach for the identification of representative/excluded patient groups may offer deeper insights in differentiating UWS from MCS in a clinically relevant setting, we included this part of the results in the Supplementary information for reference.

## Discussion

Understanding the neuropathological mechanisms underlying DOC, particularly in patients diagnosed with UWS and MCS, and developing reliable machine learning-based diagnostic tools require large, accurately labeled datasets. However, this prerequisite remains difficult to meet due to persistently high clinical misdiagnosis rates in DOC patients (Gill-Thwaites, 2006; Schnakers et al., 2009; Golden et al., 2024). While the problem of diagnostic uncertainty in DOC has been widely acknowledged, a systematic consolidation of effective, practical tools for addressing this challenge is still lacking (Porcaro et al., 2022).

In the present study, we introduce a novel data-driven strategy to identify representative patient subgroups within a larger DOC cohort by jointly considering clinical assessment scores (CRS-R) and resting-state functional connectivity features. This method identified 31 of 58 UWS patients and 23 of 30 MCS patients as representative, while the remaining 27 UWS and 7 MCS patients were scattered across the feature space without clear diagnostic separation. Validation analyses demonstrated that the classification accuracy between representative UWS and MCS patients reached 90.2% at the single-subject level (Figure 2A). In contrast, the classification accuracy for excluded UWS versus representative MCS patients, and for the entire clinically labeled dataset, dropped to 50.9 and 64.3% respectively, indicating limited diagnostic utility. Furthermore, functional connectivity differences in the DMN between representative UWS and MCS patients (Figure 4C) revealed a consistent pattern as reported in prior studies, whereas comparisons involving non-representative patients did not (Figure 4D; Supplementary Figure 2A). These findings suggest that identifying representative patients whose neuroimaging features are coherent and behaviorally consistent can substantially enhance both the accuracy and biological interpretability of machine learning models.

Our findings highlight the critical importance of data quality and label integrity in the development of machine learning models for DOC. Although neuroimaging technologies, when combined with machine learning, offer promising methods for improving diagnostic accuracy in DOC patients (Calderone et al., 2024; Velasco-Muñoz et al., 2025), these methods face significant challenges in practice. Machine learning models typically require correctly labeled, large-scale training datasets (Zhang et al., 2016; Frid-Adar et al., 2018), while small sample sizes and high misdiagnosis rates characterize most clinical neuroimaging datasets in DOC (Schnakers et al., 2009; Giacino et al., 2018; Elwell, 2023). To mitigate this issue, we proposed a principled, data-driven strategy to identify representative subgroups of patients with consistent neuroimaging and behavioral profiles. By integrating resting-state functional connectivity with CRS-R assessments, our method facilitated the construction of a high-confidence training sample space—offering a viable solution for addressing the dual challenges of small sample size and diagnostic unreliability inherent to DOC datasets. This refined sample selection not only reduced the impact of mislabeled and atypical cases, but also improved model robustness and interpretability, establishing a more trustworthy foundation for AI-based modeling in DOC.

Moreover, our study provided the interpretability of the classification results by grounding them in neurobiologically meaningful features. The DMN has been the most extensively studied intrinsic network in DOC research, with numerous studies reporting reduced functional connectivity in patients with UWS and MCS relative to healthy controls (Vanhaudenhuyse et al., 2010; Boly et al., 2012; Demertzi et al., 2015; Hannawi et al., 2015; Edlow et al., 2021; Porcaro et al., 2022). Consistent with these findings, we observed significantly diminished connectivity between PCC and both medial and lateral prefrontal cortices in representative UWS patients compared with representative MCS patients. In contrast, such disconnection patterns were absent or inconsistent when comparing excluded UWS patients to representative MCS patients. The degree and spatial extent of DMN disruption increased systematically from MCS to UWS, highlighting a gradient of network breakdown that aligns with consciousness level. These results confirm the utility of identifying representative patient subgroups: the functional connectivity contrasts between these patients are more pronounced and biologically coherent than those reported in prior studies (Vanhaudenhuyse et al., 2010; Demertzi et al., 2015), suggesting that our approach not only refines diagnostic precision but also enables more trustworthy interpretation of underlying neuropathological mechanisms.

An important assumption of our approach is that the clinical diagnosis information is more reliably represented by UWS patients with CRS-R scores ≤ 5. That is, we consider that misdiagnosis is less likely to occur in UWS patients with considerably lower CRS-R scores. The reported mean UWS CRS-R score was around 5.2–5.4 (Noé et al., 2012; Estraneo et al., 2013) and CRS-R total scores ≥ 6 may be considered as a predictor of the recovery of consciousness in UWS patients (Estraneo et al., 2013; Magliacano et al., 2023). By selecting UWS patients with CRS-R scores ≤ 5 in step 1 of our iterative refinement process, the clinical diagnosis information was integrated in our algorithm. Indeed, among the first selected 13 UWS patients with CRS-R scores ≤ 5, only two were deselected in the subsequent iterative processes.

With respect to the excluded UWS (27; 46.5% of total UWS patients) and MCS (seven; 23.3% of total MCS patients) patients, we consider that there might be two possibilities regarding their true identities. First, the most likely scenario is that the excluded patients were misdiagnosed. That is, the excluded UWS patients may actually belong to the MCS category but were misdiagnosed as UWS, and vice versa for the excluded MCS patients. This view is supported by the fact that the excluded UWS patients cannot be differentiated from the representative MCS patients (Figure 2C) and the DMN functional connectivity between the two groups showed few coherent differences (Figure 4D). Moreover, contrasting excluded MCS (*n* = 7) and representative UWS (*n* = 31) patients showed no difference at all in DMN functional connectivity (Supplementary Figure 2A). If this is the dominant fact, it is also consistent with the prior finding that a higher probability of misdiagnosis occurs with MCS being misclassified as UWS rather than the opposite (Schnakers et al., 2009).

Recent studies have increasingly questioned whether behavioral items in CRS-R truly reflect cortically mediated signs of consciousness, and these concerns align closely with the findings of representative and excluded patients identified through our refinement procedure. For example, according to the diagnostic criteria, a visual scale score of 2 (visual fixation) is considered indicative of MCS (Giacino et al., 2004). However, recent studies have argued that fixation may be mediated by subcortical activity without contribution of cortical areas, suggesting that visual fixation is likely to be more consistent with UWS (Naro et al., 2016; Naccache, 2018). Likewise, an auditory scale of 2 (auditory location), which is categorized as a UWS-level item based on CRS-R (Giacino et al., 2004), has been shown to rely on high-level cortical processing rather than simple subcortical reflexes (Carrière et al., 2020). Accordingly, recent studies have proposed that auditory location should be interpreted as a sign of MCS (Carrière et al., 2020; Pavlov et al., 2024). These findings suggest that for some DOC patients, certain behavioral diagnostic criteria of CRS-R were not consistent with the underlying neural substrates of conscious processing, potentially generating discrepancies between behavioral labels and neural signatures. Our refinement results were consistent with these results in which the identified representative patients exhibited strong behavioral-neural consistency, whereas the identified exclude patients showed clear behavioral-neural inconsistency.

## Limitations

This study has several limitations. First, the term “representative” refers to patients who exhibit high behavioral-neural consistency within our iterative, data-driven refinement framework, rather than to clinically verified gold-standard cases. The refinement results align with recent evidence suggesting that accurately labeled DOC patients should show consistency between behavioral scores and neural signatures, and that behavioral-neural inconsistency may lead to misdiagnosis (Naccache, 2018; Pavlov et al., 2024). However, due to the lack of independent validation such as longitudinal follow-up, repeated CRS-R assessments, multimodal verification with EEG or PET biomarkers, or multidisciplinary consensus evaluations, the identified representative patients in our study should be interpreted as high-consistency algorithmic samples. These cases are behaviorally and neuronally coherent and exhibit low cross-model ambiguity within the statistical structure of our dataset, but should not be regarded as definitive clinical diagnostic ones. Second, only CRS-R total scores and diagnostic labels were available in our clinical dataset (2013–2016); item-level CRS-R information was not recorded due to historical limitations in clinical documentation. Consequently, although prior studies have demonstrated that certain CRS-R items (e.g., visual fixation and auditory localization) may not reliably reflect cortically mediated signs of consciousness and therefore contribute to diagnostic inconsistencies (Naro et al., 2016; Carrière et al., 2020), the absence of item-level data in our dataset prevented us from verifying such item-specific behavioral–neural discrepancies. As a result, based on the present findings alone, we cannot determine whether specific CRS-R items require redefinition or whether revisions to the CRS-R scoring system are warranted. Moreover, a limitation common to all studies of patients with DOC is that the retrospective confirmation of behavioral misdiagnosis is challenging, if not impossible, given medical complications, examiner bias, and possible disease state transitions (e.g., some persistent UWS patients may evolve into an MCS state).

## Conclusion

In summary, we proposed a data-driven approach for identifying the representative UWS and MCS patients from a large inpatient cohort by combining clinical diagnostic information and neuroimaging biomarkers. The identified representative patients exhibited greater behavioral–neural consistency and more interpretable network-level differences than the excluded patients. These representative cases can serve as “clean templates” that were behaviorally and neuronally coherent, thus can be considered high-confidence samples for machine learning based classifier. Our results suggested that the combination of clinical and neural information could help reduce label noise in DOC datasets and provide a more coherent basis for downstream analysis and machine-learning applications in DOC classification. However, several limitations in the current study limit the interpretation and generalizability of these findings. Future studies can incorporate longitudinal CRS-R assessments, and multimodal neurophysiological validation to more rigorously evaluate behavioral–neural consistency and its potential contribution to improve diagnostic reliability in disorders of consciousness.

## Data Availability

The raw data supporting the conclusions of this article will be made available by the authors without undue reservation.

## Footnotes

1
https://afni.nimh.nih.gov/

2
https://www.fil.ion.ucl.ac.uk/spm/

## Supplementary material

SUPPLEMENTARY FIGURE 1 **(A,B)** The distribution of classification accuracy over 500 repetitions of random division of training and test sets for differentiating between all undivided UWS patients (58) and healthy control subjects (20) and between all MCS patients (30) and healthy control subjects. The average classification accuracy reached 99.5% and 97.2% in the two cases. **(C,D)** A representative example of the distribution of training and test samples in the feature space was demonstrated for classification accuracy near the average classification accuracy in each case.

SUPPLEMENTARY FIGURE 2 Two-sample *t*-test comparisons of PCC functional connectivity between identified patient groups. **(A)** Comparison between the excluded MCS patients (7) and representative UWS patients (31) showed no area of significance at the *p*-threshold. **(B,C)** Comparisons between representative and excluded MCS and UWS patients showed difference maps similar to that by comparing the representative MCS and UWS patients (Figure 4C). Of note, differences between the representative and excluded UWS patients **(C)** are more prominent because of large sample sizes (31 and 27, respectively). The significance of results is reported at *p*=0.05, corrected for multiple comparisons.
