# Supplementary figures and images for: From scales to circuits: integrating behavioral diagnosis and neural biomarkers for improved classification in disorders of consciousness
Source: Front Neurosci. 2025 Dec 18;19:1725420. doi: 10.3389/fnins.2025.1725420 (PMC12756502; doi:10.3389/fnins.2025.1725420)

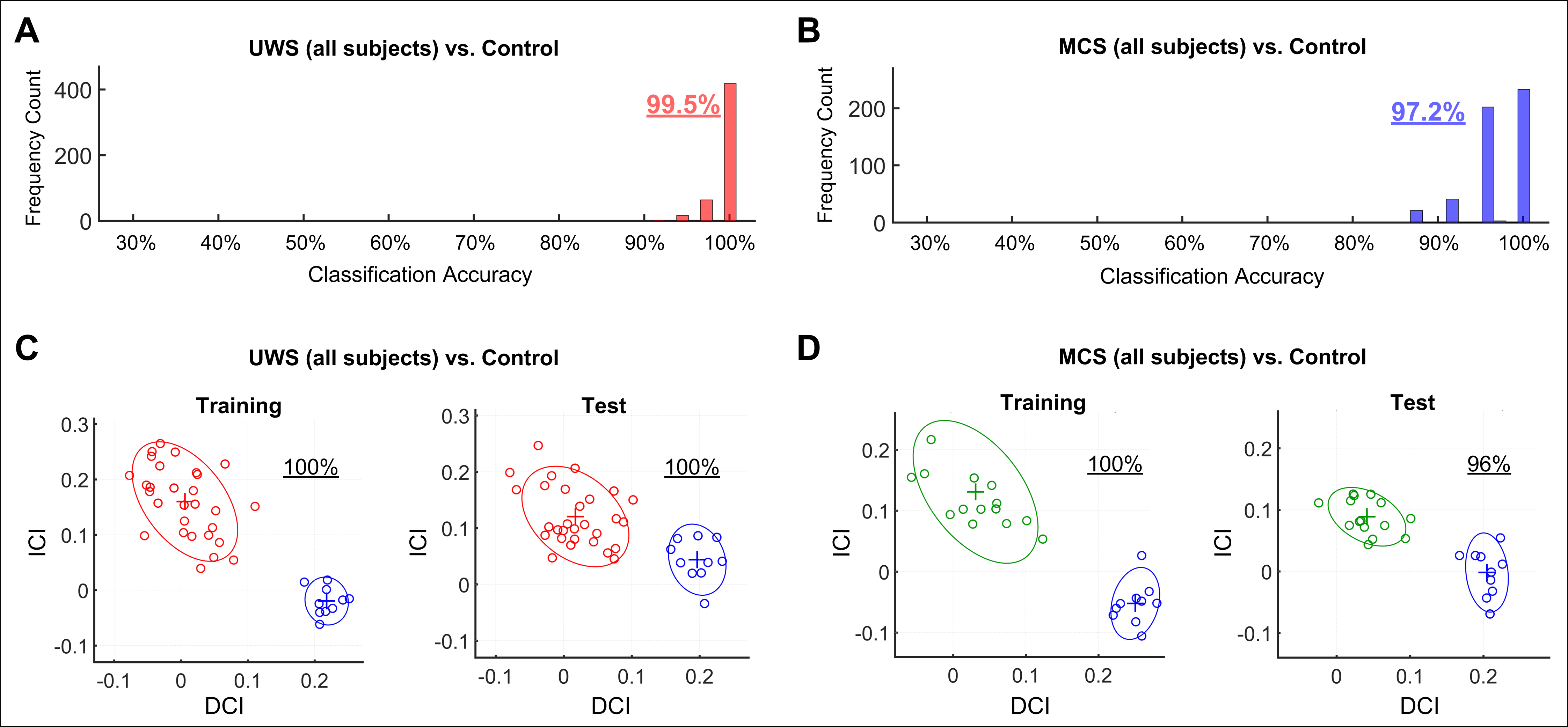

Supplement: SUPPLEMENTARY FIGURE 1 — (A,B) The distribution of classification accuracy over 500 repetitions of random division of training and test sets for differentiating between all undivided UWS patients (58) and healthy control subjects (20) and between all MCS patients (30) and healthy control subjects. The average classification accuracy reached 99.5% and 97.2% in the two cases. (C,D) A representative example of the distribution of training and test samples in the feature space was demonstrated for classification accuracy near the average classification accuracy in each case. [file Image_1.TIF]

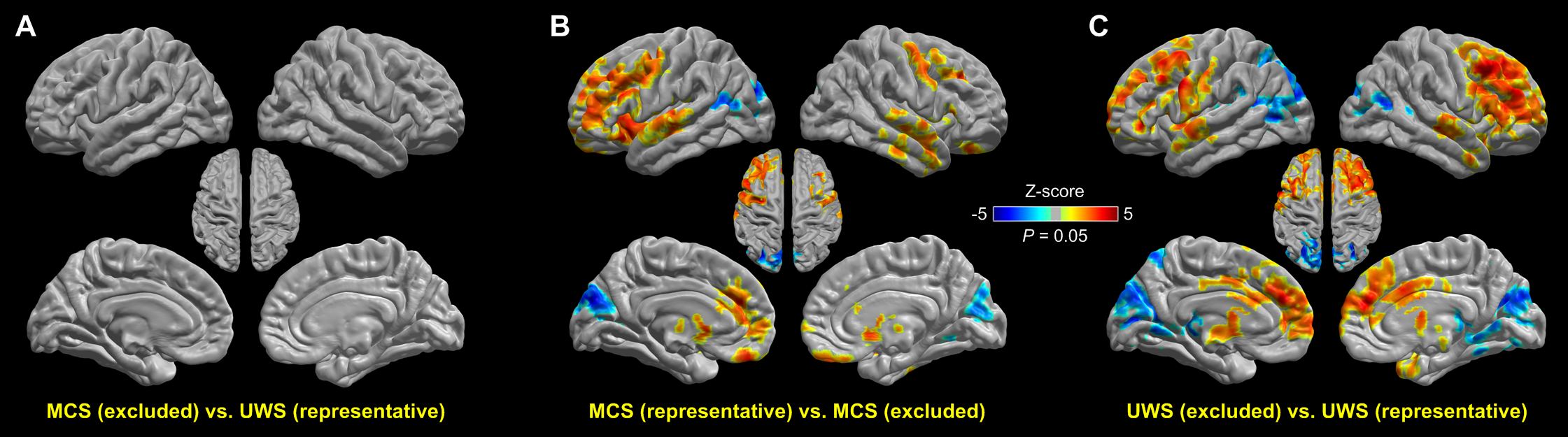

Supplement: SUPPLEMENTARY FIGURE 2 — Two-sample t-test comparisons of PCC functional connectivity between identified patient groups. (A) Comparison between the excluded MCS patients (7) and representative UWS patients (31) showed no area of significance at the p-threshold. (B,C) Comparisons between representative and excluded MCS and UWS patients showed difference maps similar to that by comparing the representative MCS and UWS patients (Figure 4C). Of note, differences between the representative and excluded UWS patients (C) are more prominent because of large sample sizes (31 and 27, respectively). The significance of results is reported at p=0.05, corrected for multiple comparisons. [file Image_2.TIF]
